# Supplementary material for: A toxin-deformation dependent inhibition mechanism in the T7SS toxin-antitoxin system of Gram-positive bacteria
Source: Nat Commun. 2022 Oct 28;13:6434. doi: 10.1038/s41467-022-34034-w (PMC9616950; doi:10.1038/s41467-022-34034-w)
Supplement: Supplementary file 1 — Supplementary Information [file 41467_2022_34034_MOESM1_ESM.pdf]

Supplementary information

A toxin-deformation dependent inhibition mechanism in the T7SS toxin-antitoxin system of Gram-positive bacteria

Yongjin Wang[1#], Yang Zhou[1#], Chaowei Shi[2#],Jiacong Liu[1], Guohua Lv[3], Huisi Huang[1], Shengrong Li[1], Liping Duan[1], Xinyi Zheng[1], Yue Liu[1], Haibo Zhou[1], Yonghua Wang[4], Zhengqiu Li[1], Ke Ding[1*], Pinghua Sun[1*], Yun Huang[5*], Xiaoyun Lu[1 *], Zhi-Min Zhang[1,6 *]

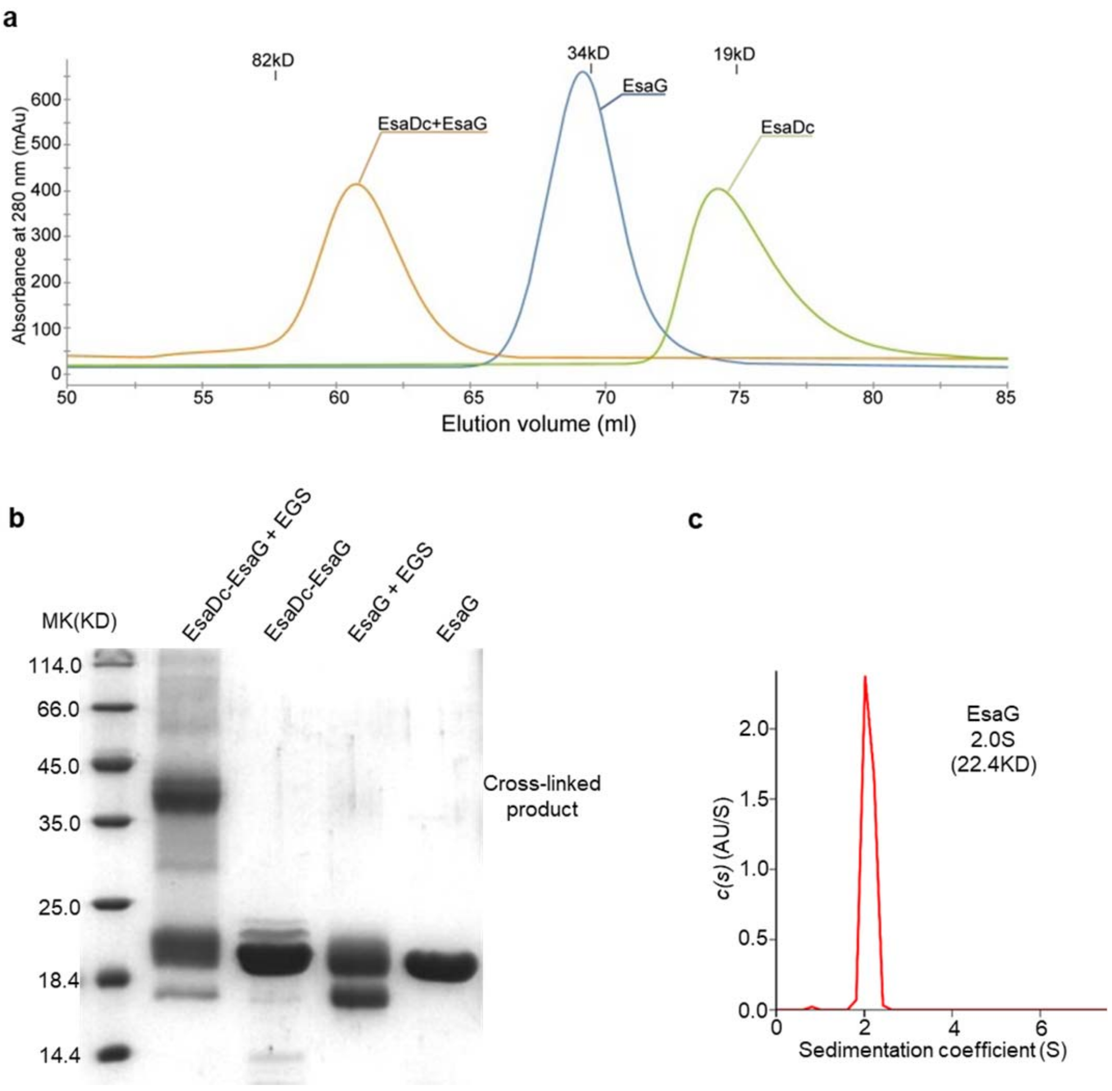

Supplementary Figure 1. EsaG proteins exist in solution as monomer. (a) Gel filtration traces of individual EsaDc, EsaG and mixed EsaDc-EsaG complex. Markers: 19 kD (Kras$_{2\text{-}169}$, Human), 34kD (TrkC$_{528\text{-}839}$, Human) and 82kD (CkTcS dimer, Kucha). **(b)** Cross-linking assay of EsaG and EsaDc-EsaG. The molar ratio of EGS: protein is set at 2.5 mM: 0.25 mM. Experiments were repeated twice with consistent results. **(c)** Density gradient centrifugation analysis of EsaG.Theoritical molecular weight of EsaG is 19.7 KD.

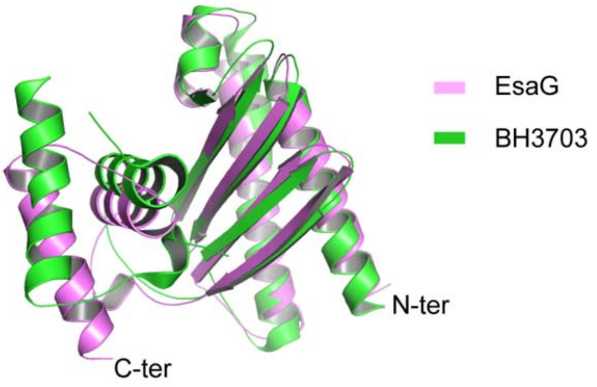

Supplementary Figure 2. Structural comparison of EsaG and BH3703.

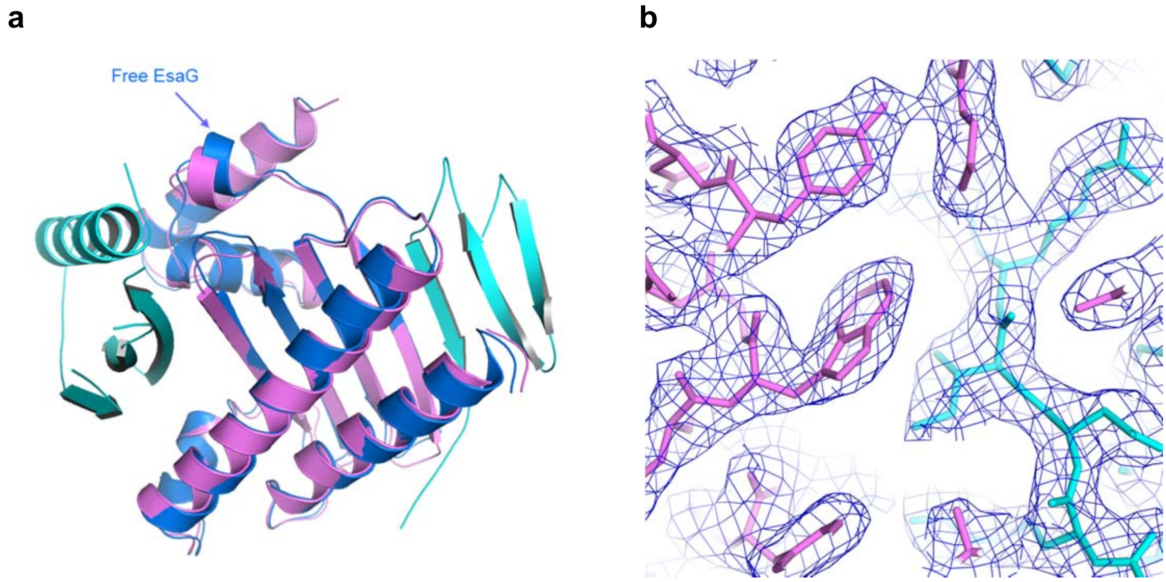

Supplementary Figure 3. Crystal structure of EsaDc-EsaG. a) Structural comparison of EsaG in EsaDc-EsaG complex and in free form. The EsaDc-EsaG complex is colored in the way as in Fig 1. Free EsaG is colored in blue. **b)** A portion of $2F_o$-F_c omit map of the structure of EsaDc-EsaG complex at a contour level of 1.0σ.

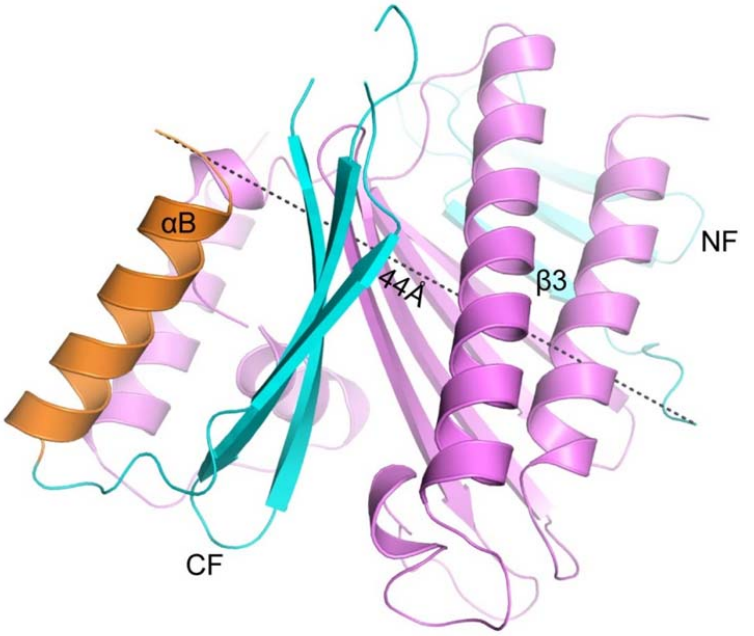

Supplementary Figure 4. Crystal structure of EsaDc-EsaG heterodimer. The straight distance between the C-terminus of NF and the N-terminus of CF is shown as black dash.

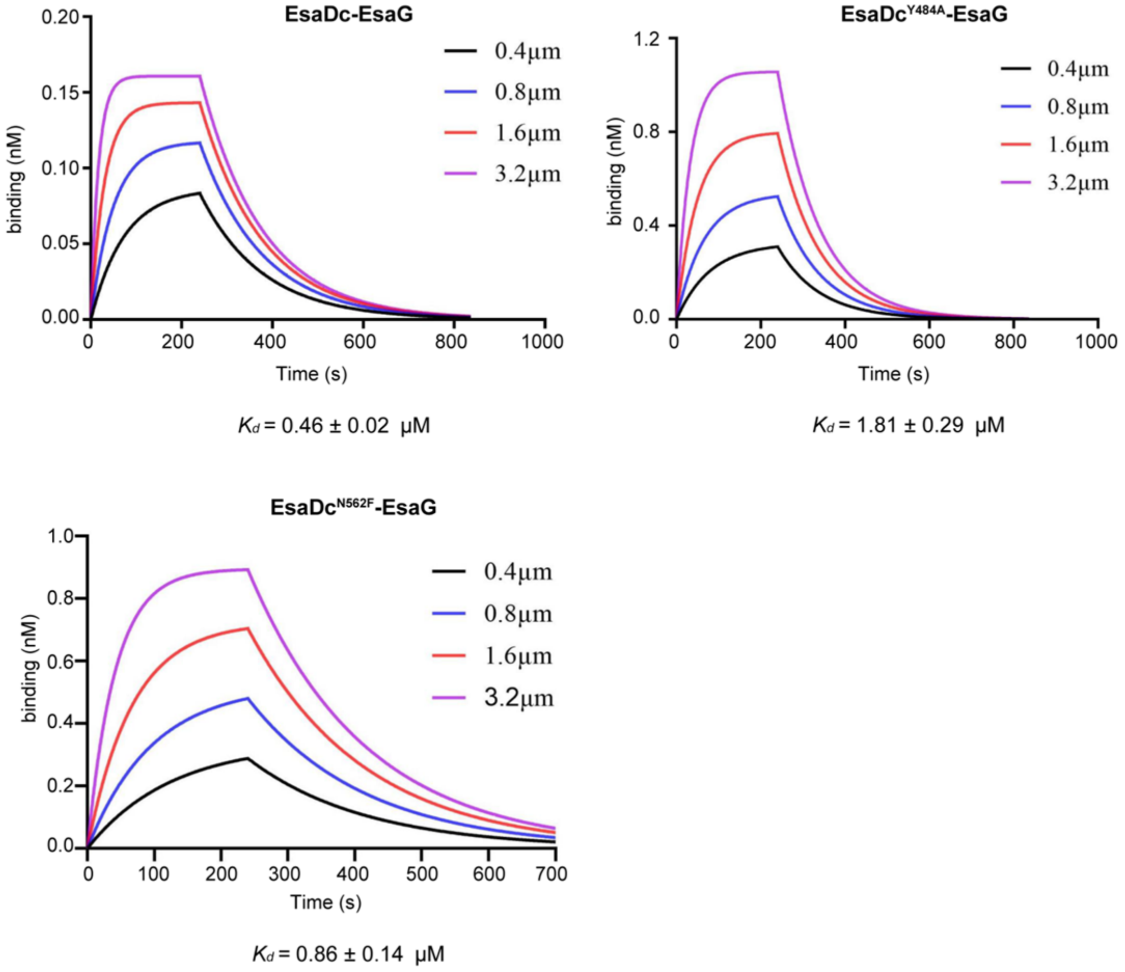

Supplementary Figure 5. Representative binding carves of EsaDc variants to EsaG examined by bio-layer interferometry assays on an OKTET K2 system (ForteBioInc., Menlo Park, CA, USA). EsaG proteins containing an N-terminal biotinylated AVI-tag were immobilized onto capture streptavidin biosensors and then exposed to different concentrations of EsaDc virants. The concentrations of EsaDc variants are labeled in each panel. Representative curves are from one of two independent experiments. The K_d value represents the mean value from the two independent experiments.

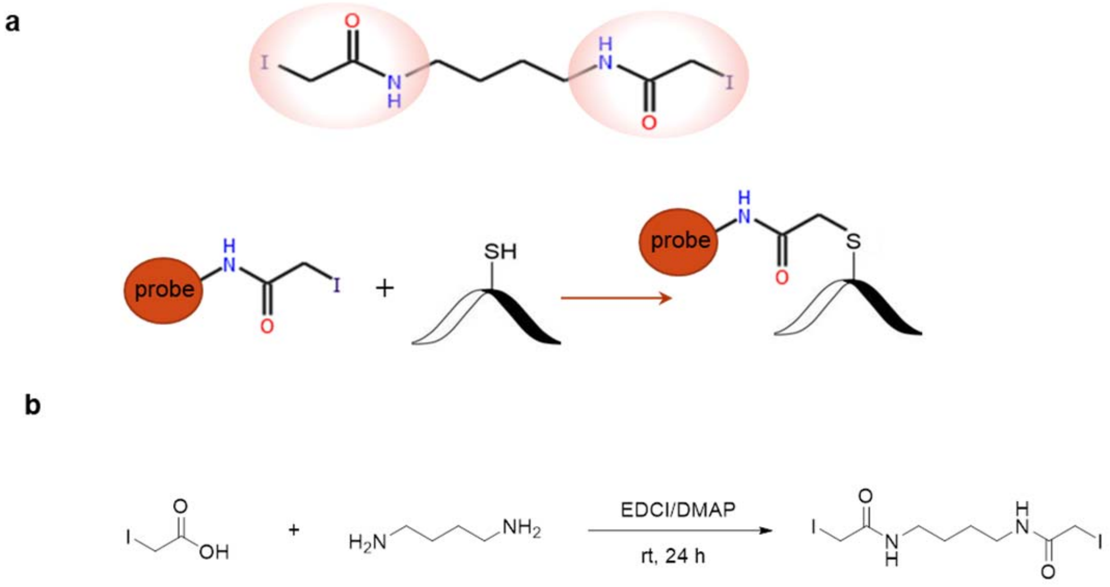

Supplementary Figure 6. Development of a cysteine-specific probe to validate the EsaDc-EsaG interaction. a) The chemical probes used for cross-linking assay. The probe contains two electrophilic iodoacetamide (IA) groups separated by a 4-carbon linker. Iodoacetamide group can react with the sulfhydryl group of cysteine side chain. b) Chemical synthesis of the the probe.

a

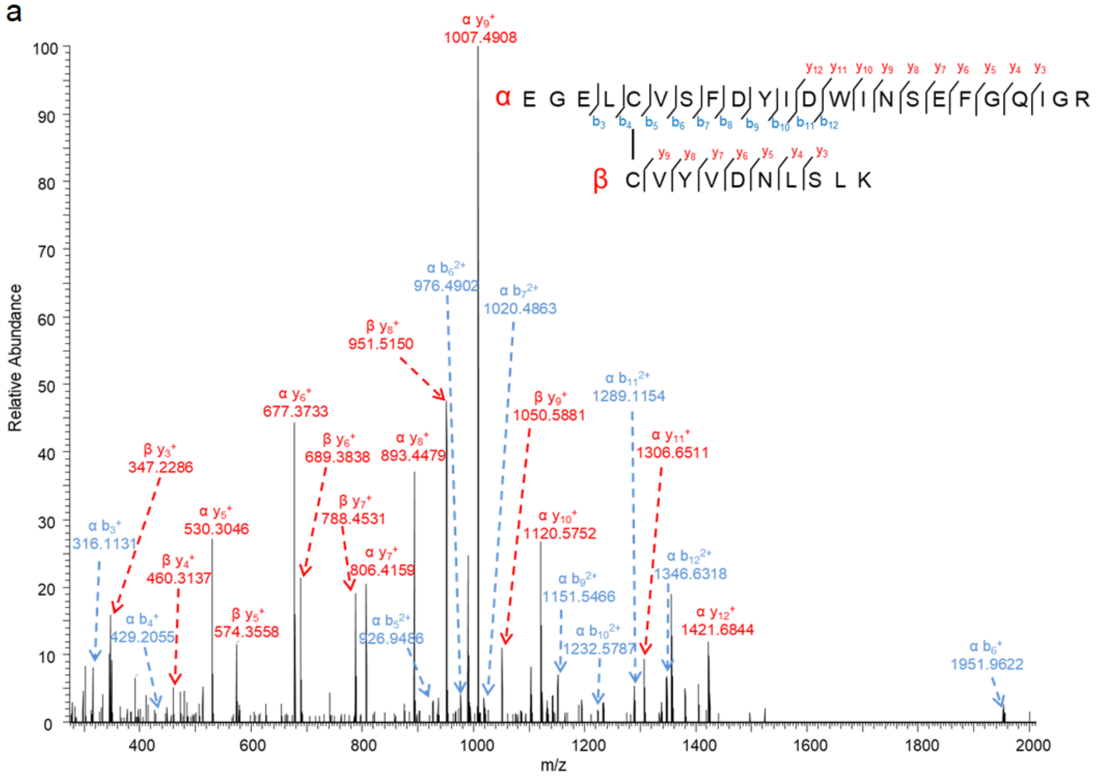

Relative Abundance
m/z
α E G E L C V S F D Y I D W I N S E F G Q I G R
β C V Y V D N L S L K
α y9+ 1007.4908
α b6 2+ 976.4902
α b7 2+ 1020.4863
β y8+ 951.5150
β y9+ 1050.5881
α b11 2+ 1289.1154
α y11+ 1306.6511
α y6+ 677.3733
β y6+ 689.3838
α y8+ 893.4479
β y3+ 347.2286
β y7+ 788.4531
α b3+ 316.1131
α y5+ 530.3046
α y10+ 1120.5752
α y7+ 806.4159
β y4+ 460.3137
α b12 2+ 1346.6318
α b9 2+ 1151.5466
α b4+ 429.2055
β y5+ 574.3558
α b5 2+ 926.9486
α b10 2+ 1232.5787
α y12+ 1421.6844
α b6+ 1951.9622

b

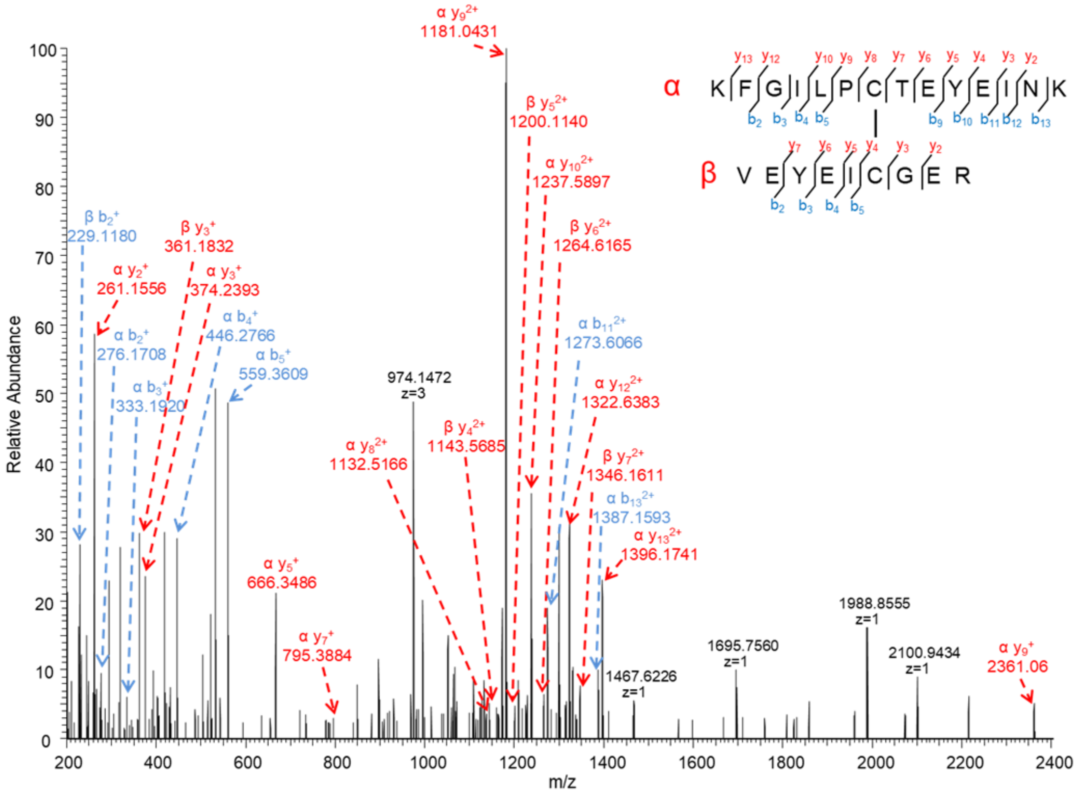

Relative Abundance
m/z
α K F G I L P C T E Y E I N K
β V E Y E I C G E R
α y9 2+ 1181.0431
β y5 2+ 1200.1140
α y10 2+ 1237.5897
β y6 2+ 1264.6165
β b2+ 229.1180
β y3+ 361.1832
α y2+ 261.1556
α y3+ 374.2393
α b4+ 446.2766
α b2+ 276.1708
α b5+ 559.3609
α b11 2+ 1273.6066
974.1472 z=3
α b3+ 333.1920
α y12 2+ 1322.6383
β y4 2+ 1143.5685
α y8 2+ 1132.5166
β y7 2+ 1346.1611
α b13 2+ 1387.1593
α y13 2+ 1396.1741
α y5+ 666.3486
1988.8555 z=1
α y7+ 795.3884
1695.7560 z=1
2100.9434 z=1
α y9+ 2361.06
1467.6226 z=1

Supplementary Figure 7. Identification of cross-linked peptide pairs of EsaDc and EsaG with AI probe. a) MS/MS analysis for the cross-linked peptide pair linking residues EsaDc-EsaG. The cross-linked product comprises amino acids 107–129 of the EsaG (α-peptide) and 494–503 of the EsaDc (β-peptide), in which EsaDc (E494C) is connected to EsaG (K111C). b) MS/MS analysis for the cross-linked peptide pair linking residues EsaDc-EsaG. The cross-linked product comprises amino acids 137–150 of the EsaG (α-peptide) and 598-606 of the EsaDc (β-peptide), in which EsaDc (N603C) is connected to EsaG (E143C). The spectrums were obtained from a 500 ng injection of corresponding cross-linked EsaDc-EsaG digest.

a

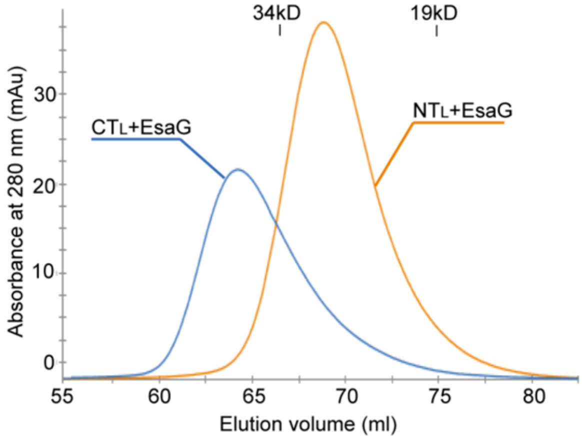

b

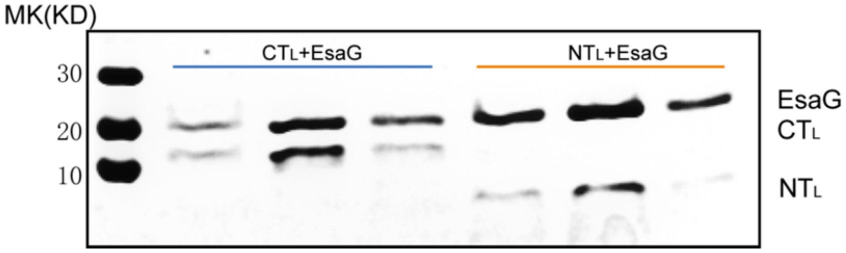

Supplementary Figure 8. Gel-filtration analysis of NF_L-EsaG complex and CF_L-EsaG complex. a) Gel filtration traces of NF_L-EsaG complex and CF_L-EsaG complex. Markers: 19kD ($Kras_{2\text{-}169}$, Human) and 34kD ($TrkC_{528\text{-}839}$, Human). b) SDS-PAGE analysis of the fractions corresponding to the OD_{280} absorbance peak of EsaG-CF_L and EsaG-NF_L, respectively. Experiments were repeated twice with consistent results.

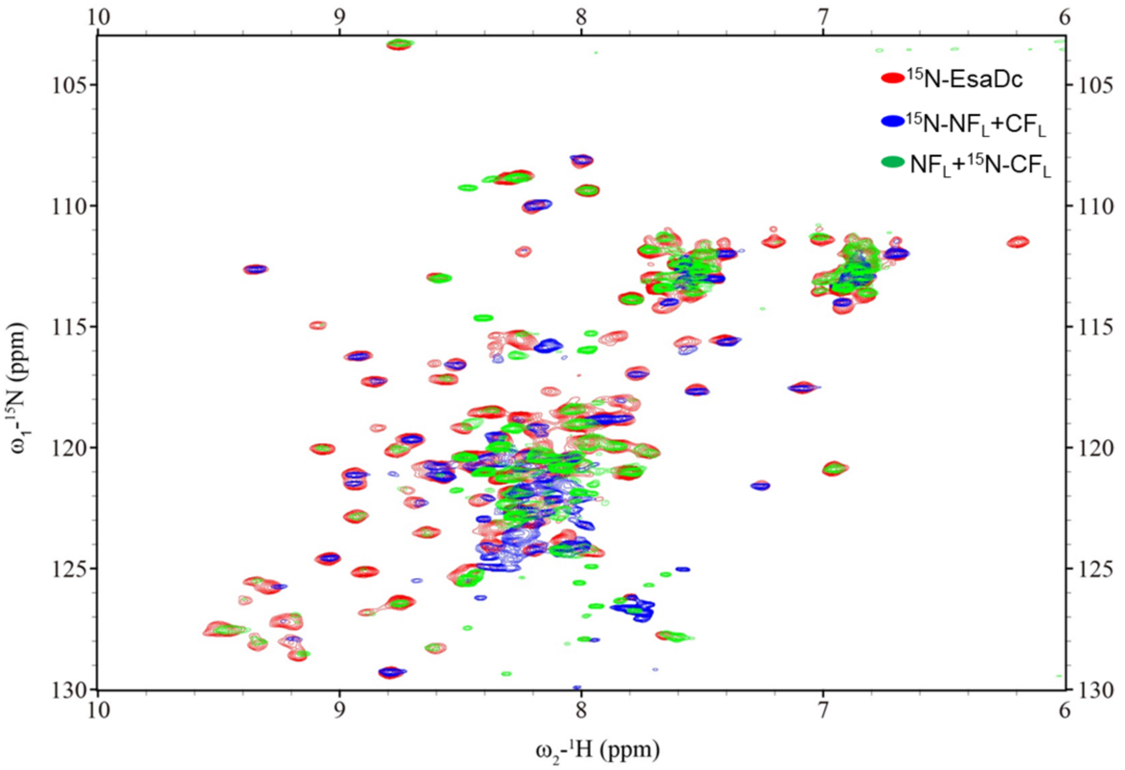

Supplementary Figure 9. Overlay of ^{1}H-^{15}N HSQC spectra for ^{15}N-labelled EsaDc (red), ^{15}N-labelled NF$_L$ in the presence of unlabeled CF$_L$ (blue) and ^{15}N-labelled CF$_L$ in the presence of unlabeled NF$_L$ (green).

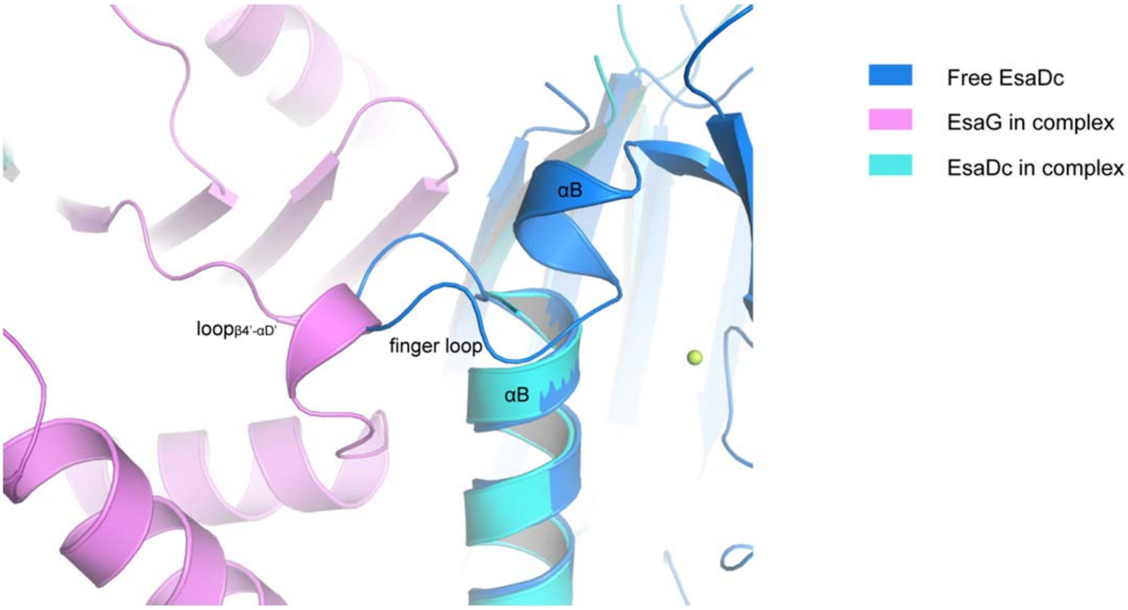

Supplementary Figure 10. Structural superposition of the free and the EsaG-bound EsaDc through their CF_L.

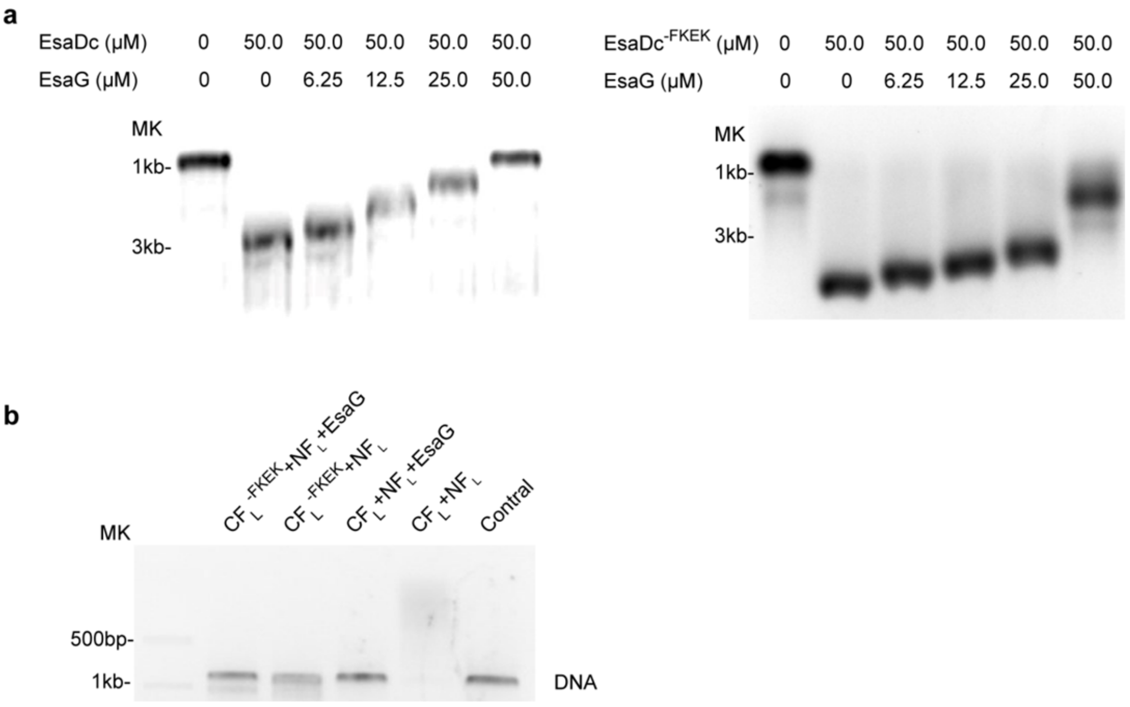

Supplementary Figure 11. The finger loop is not critical for DNA binding, but play important role for the DNase activity of EsaDc. (a) EMSA analysis of the binding between DNA and EsaDc (left)/EsaDc^{-FKEK}(right) with increasing concentrations of EsaG. Results were consistent in two independent experiments. **(b)** EMSA analysis of the DNase activity of EsaDc^{-FKEK} and the effect of EsaG. Results were consistent in two independent experiments.

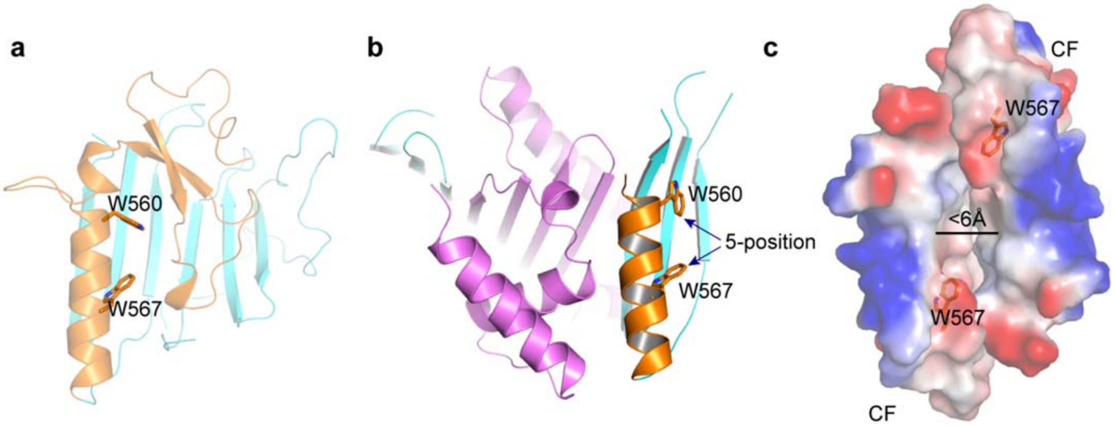

Supplementary Figure 12. Tryptophan residues in the structure of EsaDc (a), EsaDc-EsaG dimer (b) and the CF-CF interface (C). The Van der Waals diameter of the pocket in CF-CF interface is labeled in (c).

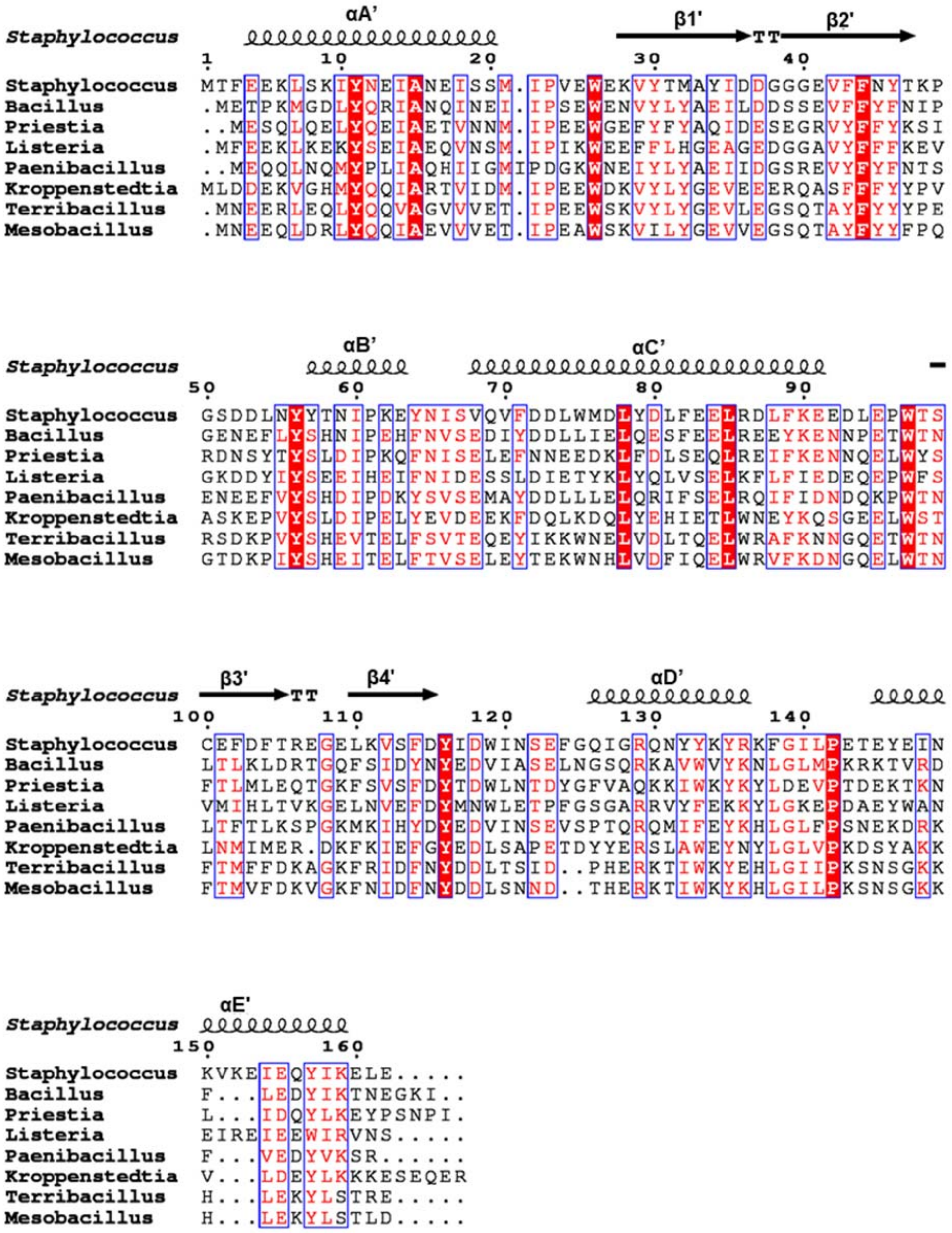

Supplementary Figure 13. Sequence alighment of EsaG-like porteins in Gram-positive bacteria. The secondary structures of EsaG are indicated above the sequence. Proteins are selected from *Staphylococcus aureus* (EsaG), *Bacillus halotolerans* (YezG), *Paenibacillus nanensis*, *Listeria grayi*, *Priestia megaterium*, *Terribacillus aidingensis*, *Mesobacillus foraminis* and *Kroppenstedtia eburnean*.Lots of Gram-positive bacteria contain more than one EsaG-like protein.

Supplementary Table 1. Data collection and refinement statistics

	Se-EsaG	EsaG (8GUP)	EsaDc (8GUN)	EsaDc-EsaG (8GUO)
**Data collection**				
Space group	P222	C2	I422	I422
Cell dimensions				
a, *b*, *c* (Å)	75.3,115.3, 160.2	90.7, 62.1, 82.8	106.6, 106.6, 151.1	111.8 111.8 171.2
α, β, γ (°)	90.00, 90.00, 90.00	90.00, 117.49, 90.00	90.00, 90.00, 90.00	90.00, 90.00, 90.00
Resolution (Å)	93.57-3.38(3.47-3.38)[a]	50.0-2.30(2.38-2.30)	30.9-2.25(2.37-2.25)	50.0-2.60(2.64-2.60)
R_{merge}	0.127(1.113)	0.217(0.413)	0.085(0.973)	0.198(0.334)
$I/\sigma(I)$	14.0(2.2)	13.5(5.6)	17.6(2.5)	31.2(7.7)
$CC_{1/2}$	0.998(0.791)	1.148(0.925)	0.998(0.737)	0.976(0.984)
Completeness (%)	99.8(99.6)	100.0(100.0)	96.0(96.7)	100.0(100.0)
Redundancy	9.7(10.1)	7.7(7.6)	10.8(10.0)	26.0(25.2)
**Refinement**				
Resolution (Å)		49.16-2.30	29.12-2.30	23.14-2.60
No. reflections		18253	20280	17104
R_{work} / R_{free}		0.216/0.254	0.186/0.230	0.208/0.225
No. atoms				
Protein		2720	2659	2101
Ligand		26	2	0
Water		118	64	125
B factors				
Protein		51.0	58.6	33.3
Ligand		95.3	41.8	0
Water		52.5	53.5	35.9
Ramachandran plot				
Fvored (%)		98.0	97.2	98.3
Allowed (%)		2.0	2.8	1.7
R.m.s.deviations				
Bond lengths (Å)		0.003	0.004	0.003
Bond angles (°)		0.57	0.69	0.63

[a]Values in parentheses are for highest-resolution shell.
