# Supplementary material for: A toxin-deformation dependent inhibition mechanism in the T7SS toxin-antitoxin system of Gram-positive bacteria
Source: Nat Commun. 2022 Oct 28;13:6434. doi: 10.1038/s41467-022-34034-w (PMC9616950; doi:10.1038/s41467-022-34034-w)
Supplement: Supplementary file 3 — Description of Additional Supplementary Files [file 41467_2022_34034_MOESM3_ESM.docx]

File Name: Supplementary Data 1

Description: Liquid chromatography-MS (LC-MS) results of cross-linking peptides to verify the EsaDc-EsaG dimer interface.

File Name: Supplementary Data 2

Description: The coordinates of the initial and final models of EsaDc-EsaG complex for MD.
