# Supplementary material for: A toxin-deformation dependent inhibition mechanism in the T7SS toxin-antitoxin system of Gram-positive bacteria
Source: Nat Commun. 2022 Oct 28;13:6434. doi: 10.1038/s41467-022-34034-w (PMC9616950; doi:10.1038/s41467-022-34034-w)
Supplement: Supplementary file 6 — Reporting Summary [file 41467_2022_34034_MOESM6_ESM.pdf]

nature portfolio

Corresponding author(s): DBPR

Last updated by author(s): Sep 14, 2022

Reporting Summary

Nature Portfolio wishes to improve the reproducibility of the work that we publish. This form provides structure for consistency and transparency in reporting. For further information on Nature Portfolio policies, see our Editorial Policies and the Editorial Policy Checklist.

Statistics

For all statistical analyses, confirm that the following items are present in the figure legend, table legend, main text, or Methods section.

n/a	Confirmed	
x	☐	The exact sample size (*n*) for each experimental group/condition, given as a discrete number and unit of measurement
x	☐	A statement on whether measurements were taken from distinct samples or whether the same sample was measured repeatedly
x	☐	The statistical test(s) used AND whether they are one- or two-sided<br>*Only common tests should be described solely by name; describe more complex techniques in the Methods section.*
x	☐	A description of all covariates tested
x	☐	A description of any assumptions or corrections, such as tests of normality and adjustment for multiple comparisons
x	☐	A full description of the statistical parameters including central tendency (e.g. means) or other basic estimates (e.g. regression coefficient) AND variation (e.g. standard deviation) or associated estimates of uncertainty (e.g. confidence intervals)
x	☐	For null hypothesis testing, the test statistic (e.g. *F*, *t*, *r*) with confidence intervals, effect sizes, degrees of freedom and *P* value noted<br>*Give P values as exact values whenever suitable.*
x	☐	For Bayesian analysis, information on the choice of priors and Markov chain Monte Carlo settings
x	☐	For hierarchical and complex designs, identification of the appropriate level for tests and full reporting of outcomes
x	☐	Estimates of effect sizes (e.g. Cohen's *d*, Pearson's *r*), indicating how they were calculated

Our web collection on statistics for biologists contains articles on many of the points above.

Software and code

Policy information about availability of computer code

Data collection	X-ray diffraction data were collected at the on the beam-line BL02U1, BL17B1 and BL19U at the Shanghai Synchrotron Radiation Facility.
Data analysis	For structural study, the HKL2000, XDS, PHENIX v1.16_3549-000, Coot v0.8.9 and Pymol v0.99 softwares were used for data processing and analysis. For cross-linking assay, pLink2 software (version 2.3.9, pFind Team, Beijing, China) were used to analyze the MS data. NMR data were processed with TOPSPIN (Bruker) and analyzed with SPARKY (Goddard and Kneller, SPARKY 3). For AUC, data analysis was performed in Sedfit. The simulations were carried out using GROMACS 2020 patched with open-source, community-developed PLUMED library, version 2.6 equipped with CHARMM36m force field for the proteins and ions, and TIP3P model for water.

For manuscripts utilizing custom algorithms or software that are central to the research but not yet described in published literature, software must be made available to editors and reviewers. We strongly encourage code deposition in a community repository (e.g. GitHub). See the Nature Portfolio guidelines for submitting code & software for further information.

Data

Policy information about availability of data

All manuscripts must include a data availability statement. This statement should provide the following information, where applicable:

- Accession codes, unique identifiers, or web links for publicly available datasets
- A description of any restrictions on data availability
- For clinical datasets or third party data, please ensure that the statement adheres to our policy

The atomic coordinates and structural factors for EsaG, the nuclease domain of EsaD and EsaG-EsaDc complex have been deposited into the PDB under the accession code 8GUN [http://doi.org/10.2210/pdb8GUN/pdb], 8GUP [http://doi.org/10.2210/pdb8GUP/pdb] and 8GUO [http://doi.org/10.2210/pdb 8GUO/pdb], respectively. The structure of BH3703 has been published in PDB under the accession code 3I0T [https://www.rcsb.org/structure/3I0T].

Human research participants

Policy information about studies involving human research participants and Sex and Gender in Research.

Reporting on sex and gender	Not involved in this study
Population characteristics	Not involved in this study
Recruitment	Not involved in this study
Ethics oversight	Not involved in this study

Note that full information on the approval of the study protocol must also be provided in the manuscript.

Field-specific reporting

Please select the one below that is the best fit for your research. If you are not sure, read the appropriate sections before making your selection.

☒ Life sciences ☐ Behavioural & social sciences ☐ Ecological, evolutionary & environmental sciences

For a reference copy of the document with all sections, see nature.com/documents/nr-reporting-summary-flat.pdf

Life sciences study design

All studies must disclose on these points even when the disclosure is negative.

Sample size	Biochemical and enzymatic assays were completed using wild type or mutants of EsaD fragments and full-length EsaG. The sample size is sufficient to delineate the mutational effects of the proteins.
Data exclusions	No data excluded
Replication	All the experiments, including chemical cross-linking, enzymatic activity test, pull-down experiments and BLI , were performed at least twice with consistent results.
Randomization	The assays performed in this study require a rational approach for activity comparison. Therefore, randomization is not applicable to our experimental set up
Blinding	Blinding is not applicable to any biochemical or cellular assay performed in this study.

Reporting for specific materials, systems and methods

We require information from authors about some types of materials, experimental systems and methods used in many studies. Here, indicate whether each material, system or method listed is relevant to your study. If you are not sure if a list item applies to your research, read the appropriate section before selecting a response.

Materials & experimental systems

n/a	Involved in the study
☒	☐ Antibodies
☒	☐ Eukaryotic cell lines
☒	☐ Palaeontology and archaeology
☒	☐ Animals and other organisms
☒	☐ Clinical data
☒	☐ Dual use research of concern

Methods

n/a	Involved in the study
☒	☐ ChIP-seq
☒	☐ Flow cytometry
☒	☐ MRI-based neuroimaging
